# Refinement of amino‐acid conformation vs. difference density maps in time‐resolved serial femtosecond crystallography data analysis

**DOI:** 10.1002/2211-5463.70250

**Published:** 2026-06-05

**Authors:** Meng Iao Fong, Yuhei Hosokawa, Lars Oliver Essen, Manuel Maestre‐Reyna

**Affiliations:** ^1^ Department of Chemistry National Taiwan University Taipei Taiwan; ^2^ Institute of Biological Chemistry Academia Sinica Taipei Taiwan; ^3^ Department of Chemistry Philipps University Marburg Germany

**Keywords:** coordinate refinement, difference density map, structural biology, time‐resolved crystallography

## Abstract

Time‐resolved crystallography is a revolutionary X‐ray diffraction technique by which the structural features of short‐lived, transient intermediates of *in crystallo* reactions can be elucidated. While visualizing time‐dependent structural changes via difference electron density maps is relatively simple, time‐resolved diffraction data is complex because it arises from a substrate‐dominated mix of the different reaction components. Thus, atomic coordinate refinement of intermediate species is challenging and prone to bias, as it requires deconvolution of the mixed‐states. To simplify the refinement process, we have developed difference electron density correlation coefficient real space refinement (dFoCC refinement). By basing coordinate refinement on comparing observed vs. calculated difference density maps, dFoCC produces reasonable atomic coordinates of intermediate species in a reproducible manner and with clearly defined quality metrics.

## Graphical abstract

The dFoCC pipeline starts with observed DED and resting‐state coordinates, which are then used to generate a library of triggered states. Correlation analysis of the calculated DED features of each candidate vs observed DED permits quantitative evaluation of candidate structural quality. Iterative cycles of candidate generation followed by correlation analysis yield highly reliable triggered state structures.

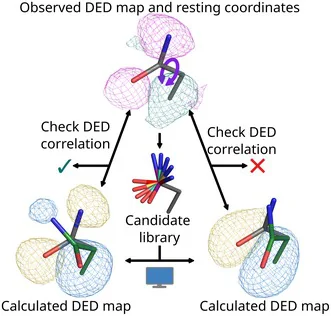

**Abbreviations**
CC: Pearson correlation coefficient
*Cr*aCRY: *Chlamydomonas reinhardtii* animal‐like cryptochrome
DED: difference electron density
dFoCC refinement: real space difference electron density correlation coefficient refinement
QY: quantum yield
RC: reaction coordinate
TR‐SFX: Time‐resolved serial femtosecond crystallography

Time‐resolved serial femtosecond crystallography (TR‐SFX) enables the structural study of intermediates [1, 2, 3] or even transitions through conical intersections [4] at atomic resolution. However, its pump‐probe setup suffers from several challenges. Specifically, limited quantum yield (QY), strong *in crystallo* attenuation, and restrictions on pump laser power to avoid multi‐photon effects generally result in low *in crystallo* activation levels [5]. Hence, triggered time‐resolved diffraction data usually consist of a mix of light‐ and dark‐adapted states, in which the latter dominates.

Conventional refinement against these mixed triggered amplitudes results in coordinates that are strongly biased toward the dark‐adapted structure. Recent methods for producing structural models describing light‐adapted intermediates based on conventional crystallographic software include occupancy refinement of light‐ and dark‐adapted coordinates [6], as well as Bayesian difference refinement [7]. Alternatively, the structure factors of the light‐adapted state can be deconvoluted from the triggered dataset via extrapolation [8]. However, these methods require accurately determining light‐adapted state occupancy in the triggered dataset, which is prone to model bias [9]. Furthermore, conventional refinement programs often rely on restraint libraries that are well‐suited for equilibrium or steady‐state experiments [10, 11], but may not be appropriate for the high energy transient reaction intermediates found in time‐resolved datasets [1, 3, 12]. In summary, structural refinement based on inaccurate occupancy information and/or inadequate restraints will produce questionable coordinates at best.

Difference electron density (DED) maps based comparing the triggered *vs*. fully dark‐adapted dataset [13] help to visualize and quantify TR‐SFX‐derived structural changes while minimizing the potential for model bias. Nonetheless, neither difference amplitudes nor DED maps are well‐suited for conventional refinement programs, because these do not generally accept negative structure factors or densities as input. Furthermore, DED maps do not encode absolute structural information, but rather only relative to the dark state. Relative structural information is also challenging to use as input for conventional refinement, although Bayesian difference refinement has partially addressed this issue [7].

Conversely, the quality of refined light‐adapted coordinates can be assessed via DED analysis [12, 14]. The correlation coefficient (CC) between the observed DED map (DED_o_) and a calculated DED map (DED_c_) based on the light‐adapted *versus* dark coordinates is used as a quality metric. This methodology has been combined with QM/MM calculations to analyze phytochrome chromophore conformational changes along pre‐defined reaction coordinates (RC) [12]. Others have coupled it to molecular dynamics simulations (MDS) for semi‐automated refinement of TR‐SFX structures [15].

In the past, we used a similar approach to drive real space difference electron density CC refinement (dFoCC, Fig. 1A). We targeted a variety of moieties, including amino acid side‐chains [2], a flavin chromophore [3], and cyclobutane pyrimidine dimer repair intermediates [3]. Our dFoCC implementation differed from previous approaches by substituting computationally costly QM/MM or MDS steps with geometrically defined RCs based on chemically favorable or geometrically sound movements [1, 2, 3, 12, 16]. In this way, dFoCC of a select few moieties complemented traditional refinement of the overall protein fold.

**Fig. 1:**
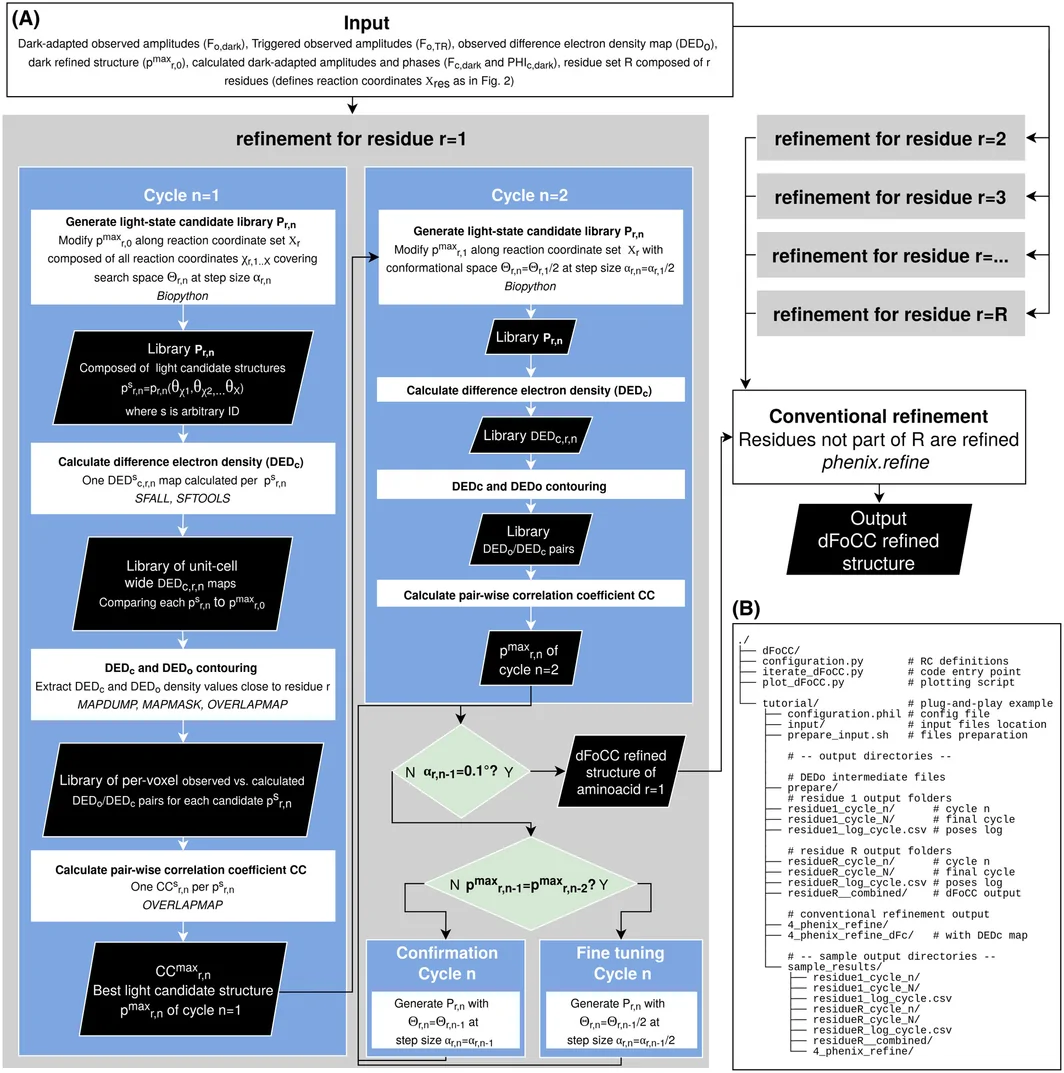
Overview of dFoCC refinement. (A) Scheme of dFoCC algorithm. Per‐residue refinement is shown as gray squares, refinement cycles as blue squares, and individual per‐cycle steps as white squares. Process outputs are represented by black parallelograms, while decisions are represented as green diamonds. Further details are provided in the main text. (B) Folder/file structure of provided dFoCC files. Cycle output folders contain best cycle poses, including DED maps and coordinate files.

In this guide, we showcase dFoCC on amino acid side‐chains, which are the most straightforward RC set to automate and package into a library (Fig. 1A). We first generate a set of possible light‐state candidate coordinates based on a well‐refined dark‐adapted structure and evaluate their quality by calculating CC between their respective DED_c_ and the experimentally observed DED_o_. This makes the process reproducible and documentable by tracking CC evolution against RC changes. Importantly, because structural quality is not evaluated via a residual target function, but based on the covariance between DED_o_ and DED_c_, our method is most sensitive to local structural changes and is robust against overestimation of occupancy [2]. As a practical example for this guide, we use TR‐SFX data from our recent publication on the photoreduction of the *Chlamydomonas reinhardtii* animal‐like cryptochrome *Cr*aCRY to refine the structural changes in four positions: H309, D321, E384, and N395 [2].

## Material

### Dependencies

- Phenix 1.20.1 [17]
- CCP4 8.0 [18]
- Coot 0.9.8 [19]
- Python 3.11
  25CB Biopython 1.85 [20]
  25CB CCTBX 2025.1 [21]
  25CB Gemmi 0.7 [22]
  25CB NumPy 1.26 [23]
  25CB pandas 2.2 [24]
  25CB SciPy 1.14 [25]
  25CB TQDM 4.67
  25CB Matplotlib 3.10 [26]

### Data

- Structural model of dark‐adapted *Cr*aCRY ($p_{r,0}^{\max}$, PDB: 8Z1J) [2] in PDB format.
- Observed and calculated amplitudes of dark‐adapted *Cr*aCRY as well as phases (F_odark_, F_cdark_, PHI_cdark_ PDB: 8Z1J) [2] in MTZ format.
- Observed structure factors of triggered *Cr*aCRY at 30 μs time delay (F_otr_, PDB: 8Z3X) [2] in MTZ format.
  25CB Using cif2mtz available in CCP4 suite [18] for file conversion produces an MTZ file with only the first block of data in the CIF file, which is the extrapolated structure factor in most cases. File conversion with cif2mtz within Gemmi (emmi cif2mtz 8z3x‐sf.cif ‐B 2) [22] results in an F_otr_ containing MTZ file by accessing the second block of the CIF file.
- A rough estimate of the occupancy of the light‐activated state in F_otr_. For *Cr*aCRY data with a delay of 30 μs. Here, we use an occupancy estimation of 18.2%, that is, *N* = 11. See tips for further information.
- A set of structure factors for final conventional refinement. Here we provide extrapolated structure factors of light‐triggered *Cr*aCRY at 30 us time delay (PDB: 8Z3X, F_ext_) [2] in MTZ format.

## Method

### File preparation

Prior to starting dFoCC refinement, input data must be copied into the input folder for the scripts to work (Fig. 1B). Below we describe additional file preparation and refer to the individual script names within the dFoCC code. The code itself is provided on https://doi.org/10.5281/zenodo.18936220. Sample scripts for each step can be found in README.md (Fig. 1B). In addition, to help the prospective user work through our showcase, we provide input files under tutorial/, as well as a condensed output under tutorial/samples_results/.

First, calculated dark‐adapted amplitudes and phases (F_cdark_ and PHI_cdark_) are calculated and stored in the same MTZ file as F_odark_. We recommend sfall within the CCP4 suite [18] to generate this file from experimental dark‐adapted amplitudes and their structural model.

Next, in order to prepare the observed difference electron density map (DED_o_), against which dFoCC will refine coordinates, The F_otr_ dataset should be scaled against F_odark_. This can be done by first combining the two datasets using cad or sftools [18], followed by scaling with scaleit [18].

Then, the DED_o_ map can be constructed with phenix.fobs_minus_fobs_map in Phenix [17] from the two scaled observed datasets and phased by the dark‐state structural model. The resulting MTZ file contains the difference amplitude dataset (dF_o_) and its associated phases (PHF_c_).

### The dFoCC algorithm

Executing python ./iterate_dFoCC.py configuration.phil initiates the algorithm shown in Fig. 1A.

The conformation of each residue r of the set R defined in the configuration.phil file (for the CraCRY data, R=H309,D321,E384,N395) is sequentially refined over a number of cycles N until a specific convergence criterium is reached (RC step size $\alpha_{r,n}$= 0.1°). During per‐residue cycle n, a library $P_{r,n}$ composed of light‐state candidate poses $p_{r,n}^{s}$ is generated in folder residue r_cycle_n/ (Fig. 1B). The superindex s denotes a unique identifier for each candidate. Candidate structures are evaluated by generating calculated difference electron density maps (DED_c_ $(p_{r,n}^{s}$)). DED_c_ $(p_{r,n}^{s}$) are then compared with DED_o_ by local pair‐wise correlation analysis. Only the pose with the highest CC, $p_{r,n}^{\max}$, is retained and is utilized in the next cycle n+1 as the initial seed for generating library $P_{r,n+1}$.

Initially, the algorithm does a rough search by having search space $\Theta_{r,n}$ cover the entire residue side‐chain conformational space, albeit with a large RC step size $\alpha_{r,n}$. However, as refinement continues, the algorithm may keep the search parameters to confirm the quality of $p_{r,n}^{\max}$ from a prior cycle. Alternatively, the search may be fine‐tuned by decreasing step size, increasing resolution at the expense of $\Theta_{r,n}$ size. Per‐residue refinement is finished when $\alpha_{r,n}$= 0.1°, a step size which is generally beyond the resolution limit of most crystal structures. The resulting dFoCC refined per‐residue pose is called $p_{r,N}^{\max}$.

At this point, $p_{r,N}^{\max}$ for all residues r of the set R are merged into a single coordinate file in folder residue*R*_combined/ (Fig. 1B), which is then subjected to conventional refinement against extrapolated amplitudes with restraints on all $p_{r,N}^{\max}$ positions. The result is the final dFoCC refined structure, which is stored in phenix_refine_dFc/ (Fig. 1B).

Below, we go into each of the algorithm steps in detail.

### Residue and point‐to‐point reaction coordinate selection

Residues to be refined with the algorithm should be selected beforehand by editing the configuration.phil file. The algorithm will automatically define RCs based on standard side‐chain dihedral angles [27] (Fig. 2A). A separate residue block must be defined for each residue of interest, as shown below.

**Fig. 2:**
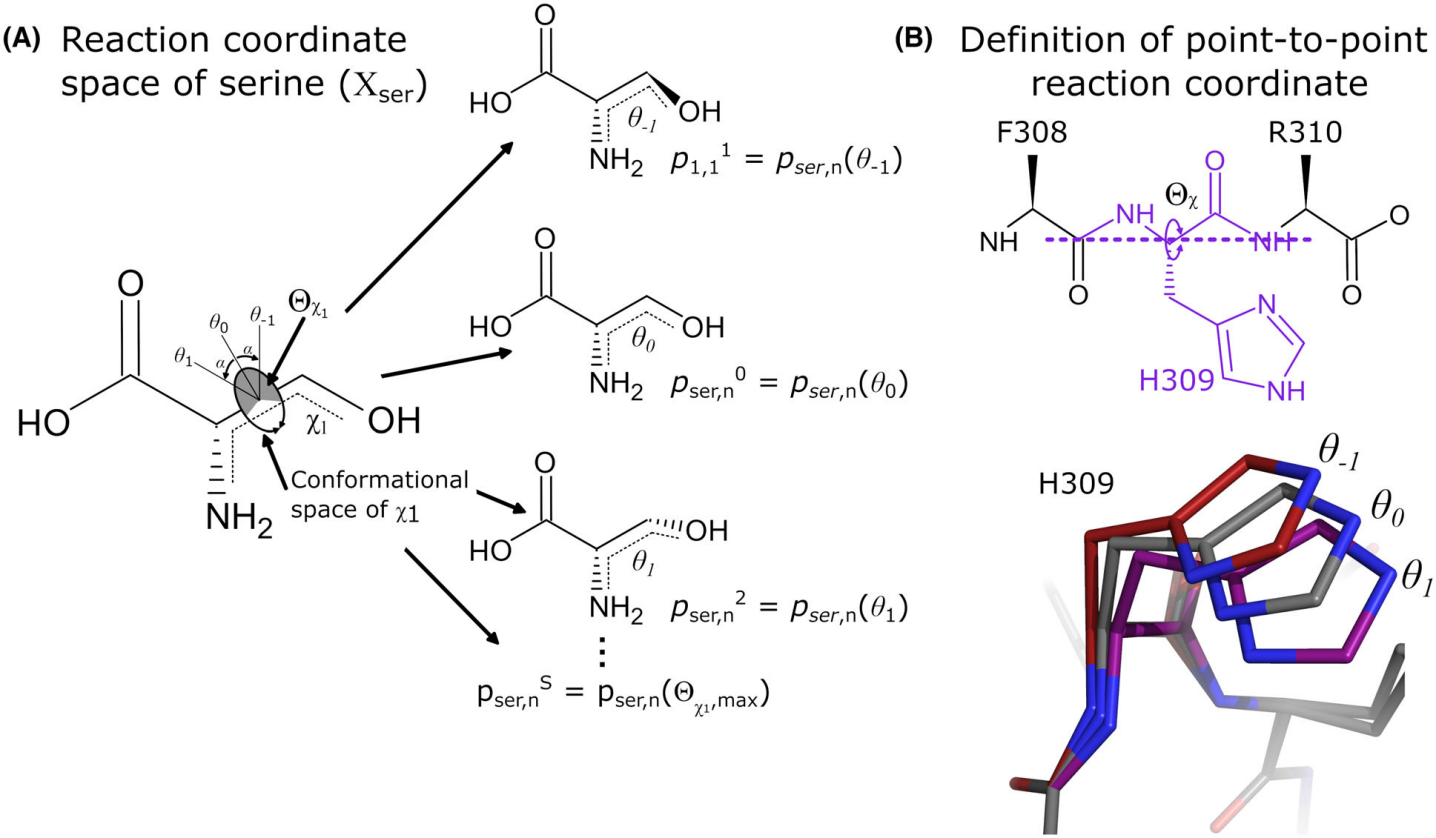
Reaction coordinates to produce the light‐state candidate library. (A) the reaction coordinate set of serine (Χ_ser_). On the left, a stick diagram of serine with its reaction coordinate χ_1_ as a dotted line, with its conformational space as a circle, within which the search space Θ_χ1_ is highlighted in gray. Here, θ_0_ corresponds to the initial position, while θ_1_ and θ_−1_ are the χ_1_ values of two modified poses. Finally, α represents the search step. On the right, the corresponding serine poses are shown. (B) Definition of point‐to‐point reaction coordinates. Top: In order to produce swiveling motions of the entire amino acid of interest, any two points along the protein main chain can be selected, which will define the rotational axis (purple dotted line). All atoms between the selected main‐chain points will be rotated (purple atoms). Bottom: example result from point‐to‐point RC for *Cr*aCRY residue H309.





residue {


 output_prefix = H309


 chain_id = A


 residue_id = 309


}





In addition, to partially take into account the coupled nature of protein main chain and side‐chain motions, users may define a custom point‐to‐point rotation axis (e.g., Fig. 2B). One such point‐to‐point RC was defined for H309 refinement (see example script below and Fig. 2B). The point‐to‐point axis is defined by two main chain atoms (restricted to N, Cα or the carbonyl C). The two selected atoms will remain fixed, while all main chain and side‐chain atoms between them will be rotated.

For a residue with this additional RC, the residue block should be further modified as exemplified below. In the algorithm, atom identification follows standard Phenix nomenclature.


residue {


 output_prefix = H309


 chain_id = A


 residue_id = 309


 main_chain_rotation {


 start {


 residue_id = 308


 atom_id = C


 }


 end {


 residue_id = 310


 atom_id = N


 }


 }


}





### Initial light‐state candidate library generation

For any given residue r and the first refinement cycle n=1, an initial library $P_{r,1}$ of light‐state candidates based on the dark‐state coordinates ($p_{r,0}^{\max})$ is generated by side‐chain atom rotation around a set of RCs ${Χ}_{r}$ defined by dihedral angles χ (Fig. 2A), as well as simple user‐defined rotations (Fig. 2B). $P_{r,1}$ is designed with search space $\Theta_{r,1}$ covering all 360° of each RCχ (Fig. 3A) with a large step size $\alpha_{r,1}$. To lower computational costs, RCs are not assigned individual Θ and α, but rather all RCs in ${Χ}_{r}$ share the same $\Theta_{r,1}$ and $\alpha_{r,1}$.

**Fig. 3:**
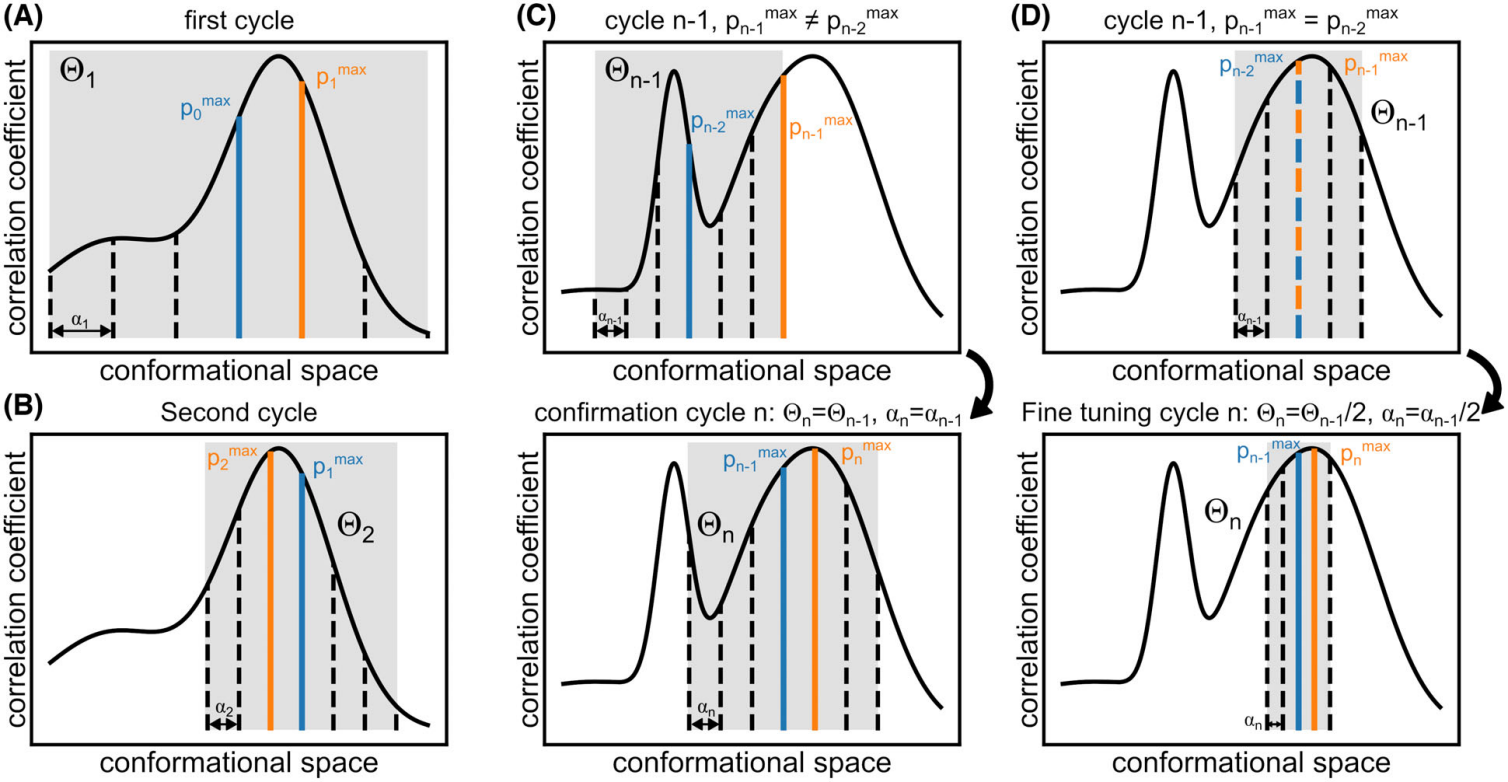
Schematics of dFoCC search algorithm. (A) Scheme of first per‐residue cycle. A black continuous curve represents the distribution of correlation coefficients over the entire conformational space, while the gray background is search space Θ_1_, and a double‐headed arrow the step size α_1_. The starting point of the search ($p_{0}^{\max}$) is shown as a blue vertical line, while the result of the first cycle as an orange line ($p_{1}^{\max}$). During the first cycle, Θ_1_ covers the entire conformational space, and α_1_ is large. (B) Scheme of second cycle, shown as in (A). Here, Θ_2_ = Θ_1_/2, and α_2_ = α_2_/2 (C) Confirmation cycle conditions, shown with the same color scheme as (A). Top: scheme of cycle *n*−1 conditions that lead to a confirmation cycle ($p_{n-1}^{\max}\neq p_{n-2}^{\max}).$ Bottom: confirmation cycle scheme. Here, Θ_*n*_ = Θ_*n*−1_, and α_*n*_ = α_*n*−1_, and the starting structure is $p_{n-1}^{\max}$. (D) Fine‐tuning cycle conditions, shown with the same color scheme as (A). Top: scheme of cycle *n*−1 conditions that lead to a confirmation cycle ($p_{n-1}^{\max}=p_{n-2}^{\max}).$ Bottom: fine‐tuning cycle scheme. Here, Θ_*n*_ = Θ_*n*−1_/2, and α_*n*_ = α_*n*−1_/2 and the starting structure is $p_{n-1}^{\max}$.

$P_{r,1}$ is thus composed of ${I_{r,\chi }}^{{Χ}_{r}}$ light‐state candidates, where $I_{r,\chi }$ is the number of steps per RC, and ${Χ}_{r}$ is the number of RCs. We set default values to ensure that ${I_{r,\chi }}^{{Χ}_{r}}$ is the largest odd number <4000 that also covers all of $\Theta_{r,1}$. To guarantee the latter, $\alpha_{r,1}$ is initially set to $360/I_{r,\chi }$.

Practically, the dFoCC algorithm uses the Bio.PDB.vectors.rotaxis2m formula of the Biopython package [20] to alter the initial dark‐state structure at residue r ($p_{r,0}^{\max}$) along all RCs in ${Χ}_{r}$, producing the initial library $P_{r,1}$ composed of ${I_{r,\chi }}^{{Χ}_{r}}$ light‐state candidates, each with a unique set of RC values $\left(\theta_{r,1,\chi =1},\theta_{r,1,\chi =2},\cdots ,\theta_{r,1,\chi ={Χ}_{r}}\right)$. To simplify the naming convention, each candidate $p_{r,1}^{s}\in P_{r,1}$ is assigned an arbitrary identifier s as a stand‐in for its defining set of RC values.

### Library generation in second and subsequent cycles

After identifying the first cycle's best candidate, $p_{r,1}^{\max}$, cycle 2 $\Theta_{r,2}$ is narrowed and the search resolution is increased by defining $\Theta_{r,2}=\Theta_{r,1}/2$ and $\alpha_{r,2}=\alpha_{r,1}/2$. Furthermore, $P_{r,2}$ is based on $p_{r,1}^{\max}$ instead of $p_{r,0}^{\max}$ (Fig. 3B). Due to the narrowing of $\Theta_{r,2}$, it is possible that poses better than $p_{r,1}^{\max}$ are left untested (Fig. 3B). To prevent this, at every cycle n>2, where $p_{r,n-1}^{\max}$ is used to generate $P_{r,n}$, the CCs of $p_{r,n-1}^{\max}$ and $p_{r,n-2}^{\max}$ are compared (Fig. 3C,D). If ${\mathrm{CC}}_{r,n-1}^{\max}>{\mathrm{CC}}_{r,n-2}^{\max}$ (Fig. 3C), then search space $\Theta_{r,n-1}$ and step size $\alpha_{r,n-1}$ may still yield better poses, and the search parameters are maintained ($\Theta_{r,n}=\Theta_{r,n-1}$, $\alpha_{r,n}=\alpha_{r,n-1}$). If, however, ${\mathrm{CC}}_{r,n-1}^{\max}={\mathrm{CC}}_{r,n-2}^{\max}$, then the resolution of search space $\Theta_{r,n-1}$ with step size $\alpha_{r,n-1}$ is insufficient to find better poses (Fig. 3D). The search parameters are then halved ($\Theta_{r,n}=\Theta_{r,n-1}/2$, $\alpha_{r,n}=\alpha_{r,n-1}/2$). We call the former a “confirmation cycle,” while the latter a “fine tuning cycle” (Fig. 1A).

### Computing DED_c_

In order to generate DED_c_ maps for each member of $P_{r,n}$, calculated amplitudes F_clight_($p_{r,n}^{s}$) are produced with sfall [18] (file. /dFoCC/difference_maps/ccp4.py), which takes F_odark_ and $p_{r,n}^{s}$ as input, and outputs F_clight_($p_{r,n}^{s}$) and PHI_clight_($p_{r,n}^{s}$). The calculated difference structure factor dF_c_($p_{r,n}^{s}$), is produced with sftools [18] (file. /dFoCC/difference_maps/ccp4.py) by subtracting F_cdark_ from F_clight_($p_{r,n}^{s}$). PHI_clight_ are discarded, because DED_c_ is constructed with PHI_cdark_ to maintain consistency with DED_o_, which was also phased with the dark‐state coordinates.

Subsequently, dF_c_($p_{r,n}^{s}$) and PHI_cdark_ are slightly modified. First, because fft [18] does not accept negative amplitudes as input, all negative dF_c_($p_{r,n}^{s}$) values are multiplied by −1, and their associated PHI_cdark_ phases are modified by π radians to produce the difference calculated phases (dPHI_c_). Next, to prevent model bias, absent amplitudes in dF_o_ are also excluded from dF_c_($p_{r,n}^{s}$). Once dF_c_($p_{r,n}^{s}$) and dPHI_c_ are prepared, DED_c_($p_{r,n}^{s}$) are computed with fft [18], resulting in unit cell‐wide 3D spaces filled with voxels with $\left(u,v,w\right)$ coordinates and DED intensity values in electron·Å^−3^ (Fig. 4 for N395, Figs S1–S3 for H309, D321, and E384, respectively).

**Fig. 4:**
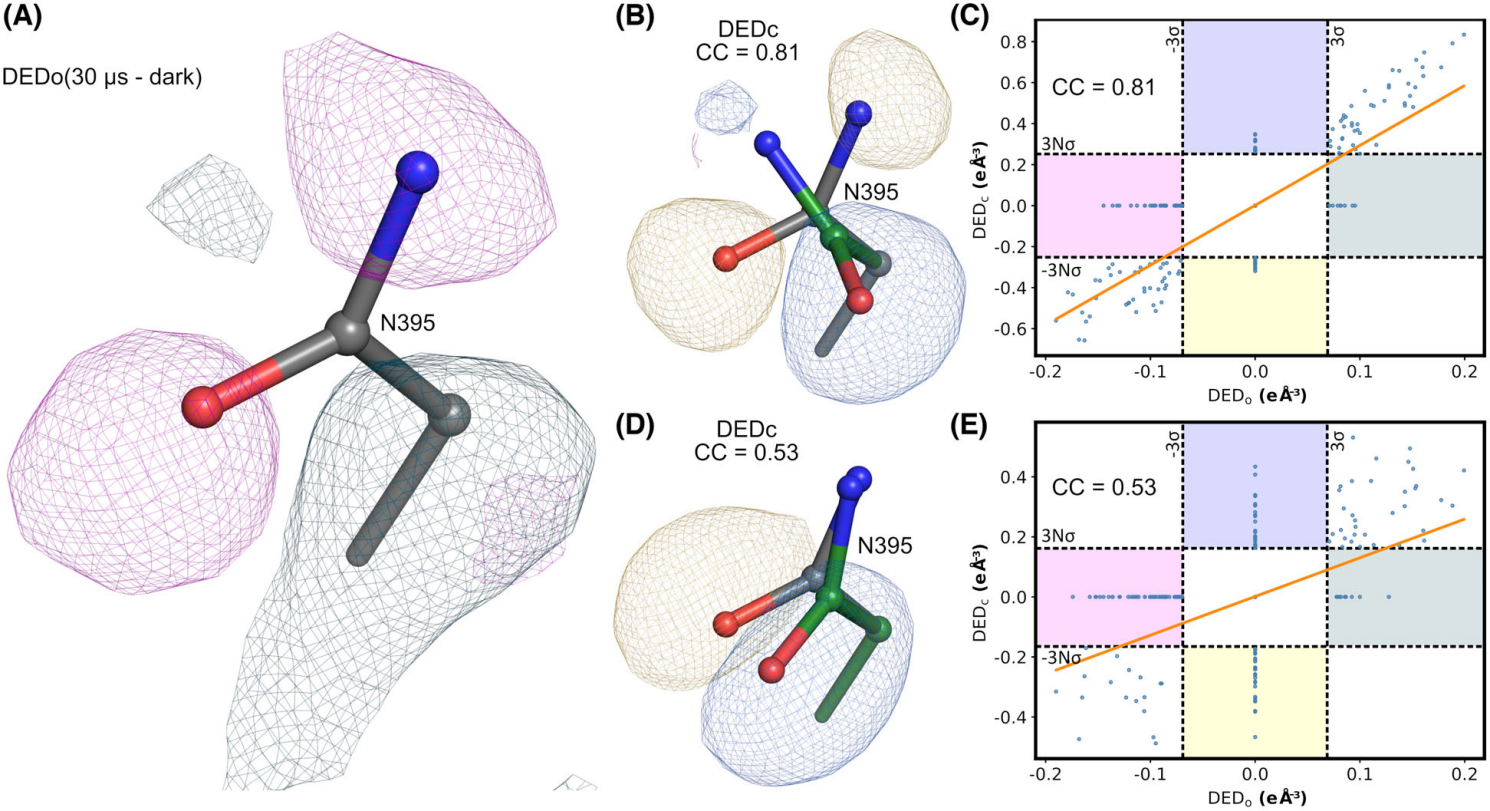
Pair‐wise correlation analysis between N395 DED_o_ and DED_c_. (A) 3σ‐contoured DED_o_ (30 μs‐dark) map superposed over the dark‐adapted conformation of *Cr*aCRY residue N395 (gray ball‐and‐stick model). Positive DED signals are shown in cyan, while negative in magenta. (B) and (C) panels correspond to a good light‐state candidate (CC = 0.81), while (D) and (E) a poor candidate (CC = 0.53). Panels (B) and (D) show the candidates' 3Nσ‐contoured DED_c_ maps, with positive signals in blue, while negative in yellow. The candidate coordinates are shown as green ball‐and‐stick models, while the dark‐adapted state in gray. Panels (C) and (E) are pair‐wise DED_o_/DED_c_ linear correlation plots in which blue dots represent individual voxel values. Significant voxels are those where both DED_c_ and DED_o_ values are outside their respective σ cutoffs (dotted lines in the graph) ($\left|{\mathrm{DED}}_{o}\left(u,v,w\right)\right|>3\sigma$, ${\mathrm{DED}}_{c}\left(u,v,w\right)>3\mathrm{N\sigma}$). There are also two types of outlier voxels. Unexplained outliers have significant DED_o_ data, but no significant DED_c_ (magenta and cyan regions). Meanwhile, the yellow and blue regions correspond to over‐modeled outliers, which only have significant DED_c_. An orange line depicts the calculated linear correlation between the DED_o_ and DED_c_ distributions. Equivalent figures for H309, D321, and E384 can be found in the (Figs S1–S3).

### DED map contouring

For the DED_o_ map, a default 3σ cutoff is applied to retain only voxels containing significant data [28]. DED_c_($p_{r,n}^{s}$) data represent the comparison between a fully light‐ *versus* a fully dark‐state (F_clight_($p_{r,n}^{s}$)‐F_cdark_). Meanwhile, DED_o_ compares the mixed triggered data vs. the dark‐adapted state (F_otr_‐F_odark_). Accordingly, DED_o_ signals are comparatively weaker than the DED_c_ ones by a factor related to light‐state occupancy. To compensate, the DED_c_ cutoff is increased by a factor of N=200%/occupancy, that is, DED_c_ maps are contoured at 3Nσ. N is typically obtained through various protocols and tools [13, 14] (see tips for options). This step is performed using MAPDUMP, MAPMASK and OVERLAPMAP [18] (file. /dFoCC/difference_maps/ccp4.py).

To perform pair‐wise CC analysis, we then extract all voxels from both DED_o_ and DED_c_ within 1.56 Å of all refined atoms in both $p_{r,n}^{s}$ and the dark‐adapted conformation $p_{r,0}^{\max}$. This extraction process is performed using phenix.map_box [17] (file. /iterate_dFoCC.py).

### Calculating pair‐wise Pearson correlation coefficient

We compare each extracted DED_c_($p_{r,n}^{s}$) map vs. the extracted DED_o_($p_{r,n}^{s}$) map by calculating their respective Pearson correlation coefficient (CC) (Fig. 4 for N395, Figs S1–S3 for H309, D321, and E384, respectively),

$$\begin{array}{l} \mathrm{CC}\left({\mathrm{DED}}_{o}\left(p_{r,n}^{s}\right),{\mathrm{DED}}_{c}\left(p_{r,n}^{s}\right)\right)=\frac{\sum_{\left(u,v,w\right)}\left({\mathrm{DED}}_{o}\left(p_{r,n}^{s}\right)-\left\langle {\mathrm{DED}}_{o}\left(p_{r,n}^{s}\right)\right\rangle \right)\left(\left({\mathrm{DED}}_{c}\left(p_{r,n}^{s}\right)-\left\langle {\mathrm{DED}}_{c}\left(p_{r,n}^{s}\right)\right\rangle \right)\right)}{\sqrt{\sum_{\left(u,v,w\right)}{\left({\mathrm{DED}}_{o}\left(p_{r,n}^{s}\right)-\left\langle {\mathrm{DED}}_{o}\left(p_{r,n}^{s}\right)\right\rangle \right)}^{2}}\sqrt{\sum_{\left(u,v,w\right)}{\left({\mathrm{DED}}_{c}\left(p_{r,n}^{s}\right)-\left\langle {\mathrm{DED}}_{c}\left(p_{r,n}^{s}\right)\right\rangle \right)}^{2}}} \end{array}$$

which can be obtained using OVERLAPMAP [18] (file. /iterate_dFoCC.py). The best light‐state candidate for cycle n ($p_{r,n}^{\max}$) is defined as the $p_{r,n}^{s}$ with the highest CC value.

### Conventional refinement on remaining residues

After sequential refinement over all residues in R, a best per‐residue pose ($p_{r,N}^{\max}$) is obtained for each of them. These are then merged into a single coordinate file stored in residue*R*_combined/ (Fig. 1B), which is then refined against an extrapolated structure factor (F_ext_) dataset. Based on the selected residue set R, the algorithm generates a phenix.refine [17] input file where residues in R are excluded from conventional refinement. All other residues undergo reciprocal space and rigid body refinement. This step is used mostly to prevent improper angles/bond distances between dFoCC refined residues and neighbors and is especially important if a point‐to‐point RC is used (Fig. 2B; Fig. S1). The result is then the fully dFoCC refined structure stored in phenix_refine_dFc/ (Fig. 1B).

## Tips and Tricks

### Visualizing results

After each cycle, a script for Coot (coot.scm) [19] is created in the cycle's working directory. To visualize the correlation of the two DED maps and the movements of the calculated structure, users may execute coot ‐s $working_dir/coot.scm. Additionally, to provide a human readable means to follow refinement, χ and CC vs. cycle plots, as well as DED_c_ vs. DED_o_ linear correlation plots (Fig. 4) are generated and stored in residue*r*_cycle_*n*/ (Fig. 1B).

### Demo mode

By running python ./iterate_dFoCC.py configuration.phil mode = demo, the algorithm skips generating $P_{r,n}$ and performs the remaining processes. The dark‐adapted structure is the default input structural model. However, users may provide another initial model or an RC list.

This mode is particularly useful in the following situations:

1. to evaluate a light‐adapted model refined by other programs by calculating CC between DED_c_ and DED_o_. This is useful to evaluate local structural fitness by CC, since global R‐factor refinement may mask the quality of refined local rearrangements.
2. to debug issues during normal execution, for example, a failure in generating the DED_c_ maps.
3. to skip initial wide range search by generating a pre‐rotated structure manually, which can then be further optimized via dFoCC refinement.

### Probe mode

Executing python ./iterate_dFoCC.py configuration.phil mode = probe produces $P_{r,1}$ and then terminates. If there is an incomplete record of normal execution, the details of the last execution determine the search parameters and RCs; otherwise, it proceeds with the initial set. This mode is useful to check if the moiety of interest approaches a reasonable position in at least one of the initial candidates, or to preserve the structural data of a given cycle. This mode is crucial when implementing custom RCs.

Alternatively, a verbose mode (python ./iterate_dFoCC.py configuration.phil verbose_mode = True) retains all intermediate files during execution, storing several gigabytes of retrievable data.

### Interruption and resumption

Upon accidental mid‐execution termination, users can resume easily by reexecuting the same command. The algorithm automatically finds the last log file and resumes from the $P_{r,n}$ generation step of the last cycle. This significantly reduces the time required to restart an aborted run.

Users can gracefully stop the process when the upcoming cycle ends by touching an empty file. /INTERRUPT. A maximum number of cycles can also be specified in the configuration file.

### Choosing an occupancy

Occupancy values can be determined via established methods [1, 2, 3, 6, 13, 14, 29] of varying accuracy, but they have been shown to be rather important to obtain a reasonable structure via extrapolation and occupancy refinement [9]. For the *Cr*aCRY example, the accurate occupancy value (18%) was obtained by analyzing negative density accumulation in extrapolated density maps at progressively lower occupancy. The method has been extensively discussed elsewhere [13].

The dFoCC algorithm is designed to maximize the covariance between local DED_c_ and DED_o_ features rather than minimize the absolute value of a residual target function. It should therefore be quite robust against inaccuracies in occupancy (Fig. 5) [2]. This is supported by the observation that dFoCC runs based on imperfect occupancy information converged to very similar coordinate sets to dFoCC refinement where an accurate occupancy value was used (Fig. 5; Fig. S4 for N395 and Figs S5–S7 for H309, D321, and E384).

**Fig. 5:**
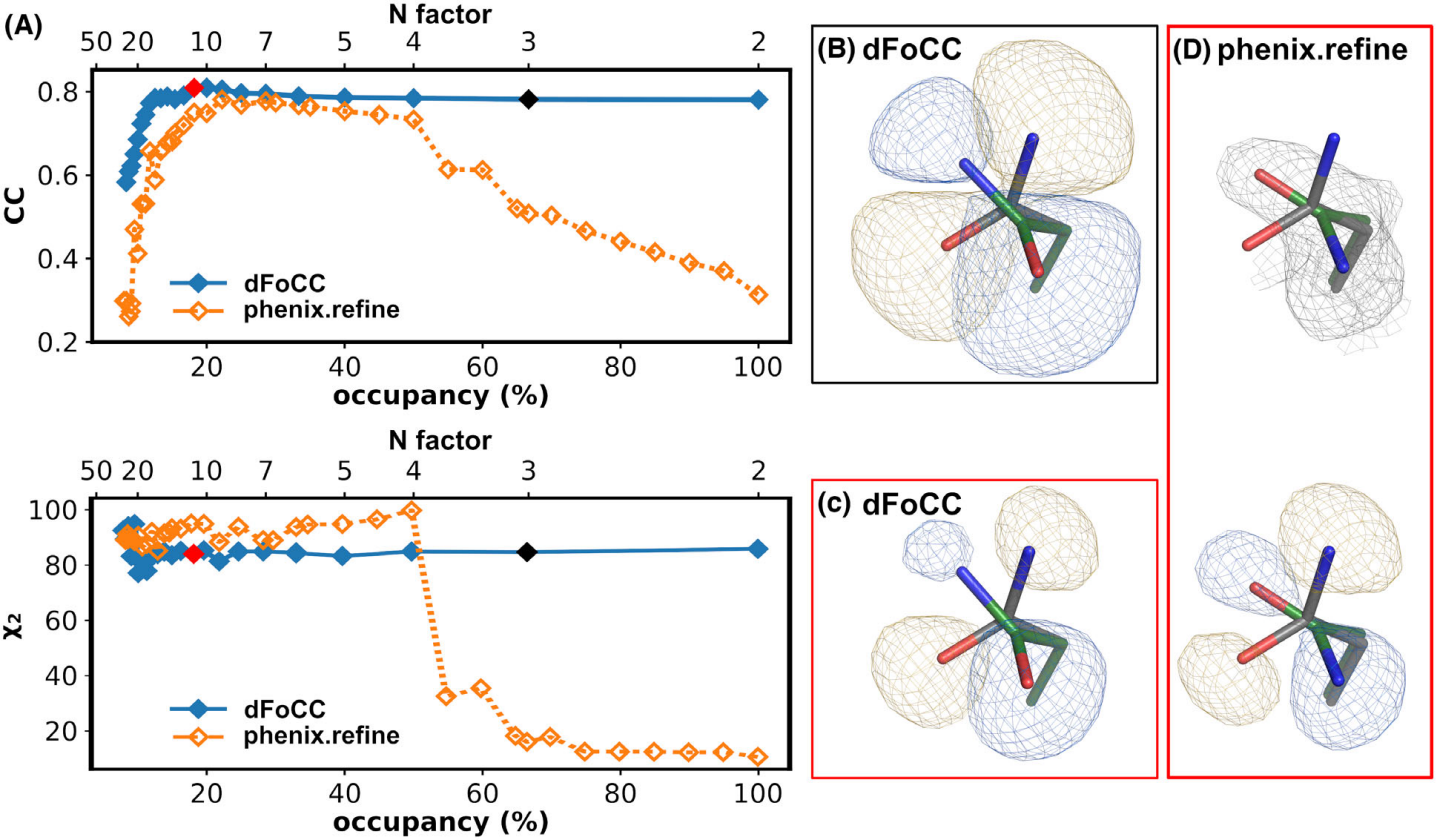
Relation between dFoCC correlation and chosen occupancy. (A) Plots comparing dFoCC vs. traditional refinement behavior. Top: CC for $p_{N395,N}^{\max}$ (y‐axis) for CraCRY N395 dFoCC or traditional refinement (blue and orange traces, respectively) at different occupancy levels (bottom x‐axis) or equivalent N‐parameter values (top x‐axis). Bottom: χ_2_ RC after dFoCC or traditional refinement (blue and orange traces, respectively) at different occupancy levels. dFoCC result for accurately estimated occupancy is highlighted in red, while based on the educated guess of the quantum yield (QY) in black. Traditional refinement was performed with phenix.refine [17] via Xtrapol8 [14], and against extrapolated structure factors. χ_1_ is reported in the Fig. S4. (B) Final DED_c_ map for educated guess dFoCC refinement (*N* = 3) superposed over dark‐adapted structure (gray) and refined model (green). (C) Final DED_c_ for accurate occupancy dFoCC refinement (*N* = 11), shown as in (B). Notice that both occupancy estimation strategies converge to the same CC value and with it, almost identical poses for N395. (D) Traditional refinement results for accurate occupancy refinement (*N* = 11). Top: extrapolated density map based on amplitudes used by phenix.refine. Bottom: Computed DED_c_ based on phenix.refine results. Equivalent figures for H309, D321, and E384 are provided in supplementary materials.

dFoCC appears to be much less sensitive to over‐ rather than underestimation (Fig. 5A; Figs S5A–S7A). For example, dFoCC refinement of residues H309, D321, E384, and N395, using *Cr*aCRY's quantum yield of 65% (N ≈ 3) as the occupancy estimation produces structures (Fig. 5B, Figs S5B to S7B) which are almost identical to the ones with the more accurate, but lower, occupancy estimation of 18% (Fig. 5C, Fig. S5C–S7C). In fact, for estimated occupancies between ~15% to 100%, dFoCC consistently converged to the same, single protein state. Meanwhile, Xtrapol8/phenix.refine‐based conventional refinement produced a variety of poses in an occupancy‐dependent manner (two for N395, three for H309 and D321, four for E384, as shown respectively in Fig. 5A, Figs S5A, S6A, S7A). The disparity of conventional refinement outcomes at the residue level produced a total of seven different, occupancy‐dependent, protein conformations.

### Troubleshooting

This algorithm was developed on a Linux machine, with an AMD Ryzen™ 97 950X CPU and 64 GB DDR5 RAM, and with Ubuntu 22.04 installed. We have tested it on the exact same machine and a Mac Mini with an Apple Silicon M4 chip. Other Unix‐like environments may also work but have not been tested.

When the error “No legitimate CC value from any structure, possibly due to overestimation of the N value” is displayed, all CC values from the structures are undefined, possibly due to all DED_o_/DED_c_ voxel pairs being outliers (Fig. 3C,E). Users can use demo mode with a random set of RCs to inspect the σ cutoff of the DED maps. Lowering the N factor may resolve the issue.

“No atom matching the ‘selection = …’ found in …” indicates that the query string of atom selection fails to select any atom in the structure file. The query string is automatically generated by default, but some edge cases might not be covered. Users may provide their own query string in the configuration file. Since the query string must follow Phenix atom selection syntax, its validity can be verified with “PDB Tools” under the “Models: Modification, minimization and dynamics” category in the Phenix suite GUI.

The algorithm may terminate mid‐execution if any of its dependencies throws an error. This is usually caused by atom collision, because no steric clash filtering is implemented in the current version of the algorithm. Users are expected to check the error logs, execute probe mode or demo mode with the exact RC set that caused the error, and adjust the initial RC set/range to avoid the collision. However, automated steric clash filtering will be introduced in future versions of dFoCC.

Advanced users should be very cautious when establishing custom RCs for their systems. The user should not rely solely on automatically generated metrics, such as CC, which may still be maximized despite the wrong set of RCs. Instead, chemical understanding of the system at hand must be the guiding principle when evaluating candidate RCs. On a case‐by‐case basis, occupancy analysis and inspection of candidate libraries via probe mode will be helpful. Finally, the user must be willing to accept that some subtle structural changes may not be amenable to a semi‐automated approach. Then, only extremely careful occupancy determination, followed by refinement against extrapolated structure factors with manually weakened or even switched‐off selected restraints may yield reasonable coordinates [13].

## Discussion

The dFoCC algorithm has proven to be a powerful tool in our hands to refine local structural changes in a select subset of chemical entities [2, 3]. Once RCs have been defined for the target system, such as the current example of side‐chain rotation, dFoCC refinement produces reproducible coordinates with a clearly defined quality parameter, the DED_o_ vs. DED_c_ correlation coefficient.

Regarding computational costs and human input, dFoCC suffers from many of the same limitations as MDS‐based refinement [15], although we suggest to a lesser degree. First, and much like MDS force‐field approaches, dFoCC‐based modeling of standard amino acid side‐chains is relatively easy due to their clearly defined rotational RCs. However, refinement of non‐standard residues, such as chromophores or enzyme substrates, is not trivial. Similarly to parameterization in MDS, the user must set up RCs that adequately describe the chemical and structural changes undergone by these special moieties. In the past, we have done so for flavins and pyrimidine cyclobutanes [3]. Although their RCs are not included in the current package, they are described in detail in their respective publications and can be readily implemented by a user. In the future, we aim to provide a fully‐fledged software suite that allows plug‐and‐play functionality for the most common groups of chromophores, such as flavins, p‐coumaric acid, and retinol derivatives.

Secondly, dFoCC is more computationally expensive than traditional refinement (9.5 h of computing time for the current showcase on an AMD Ryzen 7950X CPU *vs*. 8 min for a single run of refinement by phenix.refine v1.20.1–4487). However, dozens of phenix.refine runs are typically necessary for refinement to converge, while usually only one dFoCC run is needed. Compared to MDS or QM/MM approaches, where full trajectories can take several days to compute and weeks to analyze, dFoCC is significantly faster. Even when compared to the “slow and careful” approach of Xtrapol8 [14], refinement times are comparable (15 h for 45 occupancies on an AMD Ryzen 7950X CPU). dFoCC refinement results, with the exception of D321 (Fig. S6), clearly adjust better to the experimental DED and extrapolated maps (Fig. 5, Figs S5 and S7).

Another important issue is the limited ability of the showcase's RCs to model large‐scale conformational changes involving many residues simultaneously. In fact, our point‐to‐point RC successfully followed coupled main‐chain swiveling and side‐chain rotation of residues D321 and H309 (Fig. 2B, Fig. S6). For D321 and within the range of occupancies where Xtrapol8 and dFoCC results converged, the former yielded better results (Fig. S6). Nevertheless, it should be possible to expand dFoCC functionality in the future by introducing general refinement RCs, for example via principal component analysis of MDS trajectories [30]. However, the significance of DED maps is limited to situations where structural changes remain relatively minor [28]. Thus, being restricted to local effects may be inherent to the DED approach, rather than to RC generation. In good agreement, the vast majority of structural changes studied via TR‐SFX have not included dramatic main‐chain motions [2, 3, 8, 31, 32, 33].

Due to these limitations, dFoCC should not be envisioned as a substitute for traditional refinement but as a complementary approach to accurately follow local structural changes of a few crucial moieties, which are then propagated through the protein structure via a final round of phenix.refine reciprocal space refinement (Fig. 1A). In conclusion, despite its current shortcomings, we propose that dFoCC is a valuable tool for analysis of local structural changes derived from TR‐SFX studies and hope that prospective users find this guide useful.

## Conflict of interest

The authors declare no conflict of interest.

## Author contributions

MIF, YH, LOE, and MMR coded and tested the dFoCC algorithm. MIF, YH, and MMR analyzed data. MIF and MMR wrote the manuscript. MIF, YH, LOE, and MMR edited the manuscript. MMR conceived the research.

## Supporting information


**Fig. S1.** Pair‐wise correlation analysis between H309 DED_o_ and DED_c_.
**Fig. S2.** Pair‐wise correlation analysis between D321 DED_o_ and DED_c_.
**Fig. S3.** Pair‐wise correlation analysis between E384 DED_o_ and DED_c_.
**Fig. S4.** N395 χ_1_ dihedral angle values vs. estimated occupancy during dFoCC (blue trace) and traditional refinement (orange trace).
**Fig. S5.** Relation between dFoCC correlation and chosen occupancy for H309.
**Fig. S6.** Relation between dFoCC correlation and chosen occupancy for D321.
**Fig. S7.** Relation between dFoCC correlation and chosen occupancy for E384.

## Data Availability

All dark‐adapted and light‐triggered coordinate and amplitude files are available on the Protein Data Bank under the PDB code 8Z1J and 8Z3X, respectively. The scripts of the whole algorithm can be retrieved from https://doi.org/10.5281/zenodo.18936220. The most up‐to‐date version can be obtained from https://github.com/ntu‐mmr‐lab/dFoCC/tree/main.
