# Supplementary material for: Refinement of amino‐acid conformation vs. difference density maps in time‐resolved serial femtosecond crystallography data analysis
Source: FEBS Open Bio. 2026 Jun 5;16(10):1899–910. doi: 10.1002/2211-5463.70250 (PMC13398853; doi:10.1002/2211-5463.70250)
Supplement: Supplementary file 1 [file FEB4-16-1899-s001.docx]

Supplementary Figure 1 Pair-wise correlation analysis between H309 DED_o_ and DED_c_. (A) 3σ-contoured DED_o_ (30 µs -dark) map superposed over the dark-adapted conformation of CraCRY residue H309 (grey ball-and-stick model). Positive DED signals are shown in cyan, while negative in magenta. (B) and (C) panels correspond to a good light-state candidate (CC = 0.72), while (D) and (E) a poor candidate (CC = 0.23). Panels (B) and (D) show the candidates’ 3Nσ-contoured DED_c_ maps, with positive signals in blue, while negative in yellow. The candidate coordinates are shown as green ball-and-stick models, while the dark-adapted state in gray. Panels (C) and (E) are pairwise DED_o_/DED_c_ linear correlation plots in which blue dots represent individual voxel values. Significant voxels are those where both DED_c_ and DED_o_ values are outside their respective σ cutoffs (dotted lines in the graph) (|DED_o_(u,v,w)|>3σ, DED_c_(u,v,w)>3Nσ). There are also two types of outlier voxels. Unexplained outliers have significant DED_o_ data, but no significant DED_c_ (magenta and cyan regions). Meanwhile, the yellow and blue regions correspond to over-modelled outliers, which only have significant DED_c_. An orange line depicts the calculated linear correlation between the DED_o_ and DED_c_ distributions.

Supplementary Figure 2 Pair-wise correlation analysis between D321 DED_o_ and DED_c_. (A) 3σ-contoured DED_o_ (30 µs -dark) map superposed over the dark-adapted conformation of CraCRY residue D321 (grey ball-and-stick model). Positive DED signals are shown in cyan, while negative in magenta. (B) and (C) panels correspond to a good light-state candidate (CC = 0.70), while (D) and (E) a poor candidate (CC = 0.31). Panels (B) and (D) show the candidates’ 3Nσ-contoured DED_c_ maps, with positive signals in blue, while negative in yellow. The candidate coordinates are shown as green ball-and-stick models, while the dark-adapted state in gray. Panels (C) and (E) are pairwise DED_o_/DED_c_ linear correlation plots in which blue dots represent individual voxel values. Significant voxels are those where both DED_c_ and DED_o_ values are outside their respective σ cutoffs (dotted lines in the graph) (|DED_o_(u,v,w)|>3σ, DED_c_(u,v,w)>3Nσ). There are also two types of outlier voxels. Unexplained outliers have significant DED_o_ data, but no significant DED_c_ (magenta and cyan regions). Meanwhile, the yellow and blue regions correspond to over-modelled outliers, which only have significant DED_c_. An orange line depicts the calculated linear correlation between the DED_o_ and DED_c_ distributions.

Supplementary Figure 3 Pair-wise correlation analysis between E384 DED_o_ and DED_c_. (A) 3σ-contoured DED_o_ (30 µs -dark) map superposed over the dark-adapted conformation of CraCRY residue H309 (grey ball-and-stick model). Positive DED signals are shown in cyan, while negative in magenta. (B) and (C) panels correspond to a good light-state candidate (CC = 0.47), while (D) and (E) a poor candidate (CC = 0.18). Panels (B) and (D) show the candidates’ 3Nσ-contoured DED_c_ maps, with positive signals in blue, while negative in yellow. The candidate coordinates are shown as green ball-and-stick models, while the dark-adapted state in gray. Panels (C) and (E) are pairwise DED_o_/DED_c_ linear correlation plots in which blue dots represent individual voxel values. Significant voxels are those where both DED_c_ and DED_o_ values are outside their respective σ cutoffs (dotted lines in the graph) (|DED_o_ (u,v,w)|>3σ, DED_c_ (u,v,w)>3Nσ). There are also two types of outlier voxels. Unexplained outliers have significant DED_o_ data, but no significant DED_c_ (magenta and cyan regions). Meanwhile, the yellow and blue regions correspond to over-modelled outliers, which only have significant DED_c_. An orange line depicts the calculated linear correlation between the DED_o_ and DED_c_ distributions.

Supplementary Figure 4 N395 χ_1_ dihedral angle values vs. estimated occupancy during dFoCC (blue trace) and traditional refinement (orange trace). Traditional refinement was performed with phenix.refine via Xtrapol8, and against extrapolated structure factors. χ_2_ is reported in the main text Fig. 5.

Supplementary Figure 5 Relation between dFoCC correlation and chosen occupancy for H309. (A) Plots comparing dFoCC vs. traditional refinement behavior. Top: CC for $p_{H309,N}^{max}$ (y-axis) for CraCRY H309 dFoCC or traditional refinement (blue and orange traces, respectively) at different occupancy levels (bottom x-axis) or equivalent N-parameter values (top x-axis). Middle: χ_1_ RC after dFoCC or traditional refinement (blue and orange traces, respectively) at different occupancy levels. Bottom: χ_2_ RC after dFoCC or traditional refinement (blue and orange traces, respectively) at different occupancy levels. dFoCC result for accurately estimated occupancy is highlighted in red, while based on the educated guess of the quantum yield (QY) in black. Traditional refinement was performed with phenix.refine via Xtrapol8, and against extrapolated structure factors. (B) Final DED_c_ map for educated guess dFoCC refinement (N=3) superposed over dark-adapted structure (grey) and refined model (green). (C) Final DED_c_ for accurate occupancy dFoCC refinement (N=11), shown as in (B). Notice that both occupancy estimation strategies converge to the same CC value and with it, almost identical poses for H309. (D) Traditional refinement results for accurate occupancy refinement (N=11). Top: Computed DED_c_ based on phenix.refine results. Bottom: extrapolated density map based on amplitudes used by phenix.refine.

Supplementary Figure 6 Relation between dFoCC correlation and chosen occupancy for D321. (A) Plots comparing dFoCC vs. traditional refinement behavior. Top: CC for $p_{D321,N}^{max}$ (y-axis) for CraCRY D321 dFoCC or traditional refinement (blue and orange traces, respectively) at different occupancy levels (bottom x-axis) or equivalent N-parameter values (top x-axis). Middle: χ_1_ RC after dFoCC or traditional refinement (blue and orange traces, respectively) at different occupancy levels. Bottom: χ_2_ RC after dFoCC or traditional refinement (blue and orange traces, respectively) at different occupancy levels. dFoCC result for accurately estimated occupancy is highlighted in red, while based on the educated guess of the quantum yield (QY) in black. Traditional refinement was performed with phenix.refine via Xtrapol8, and against extrapolated structure factors. (B) Final DED_c_ map for educated guess dFoCC refinement (N=3) superposed over dark-adapted structure (grey) and refined model (green). (C) Final DED_c_ for accurate occupancy dFoCC refinement (N=11), shown as in (B). Notice that both occupancy estimation strategies converge to the same CC value and with it, almost identical poses for D321. (D) Traditional refinement results for accurate occupancy refinement (N=11). Top: Computed DED_c_ based on phenix.refine results. Bottom: extrapolated density map based on amplitudes used by phenix.refine.

Supplementary Figure 7 Relation between dFoCC correlation and chosen occupancy for E384. (A) Plots comparing dFoCC vs. traditional refinement behavior. Top left: CC for $p_{E384,N}^{max}$ (y-axis) for CraCRY E384 dFoCC or traditional refinement (blue and orange traces, respectively) at different occupancy levels (bottom x-axis) or equivalent N-parameter values (top x-axis). Top right: χ_1_ RC after dFoCC or traditional refinement (blue and orange traces, respectively) at different occupancy levels. Bottom left: χ_2_ RC after dFoCC or traditional refinement (blue and orange traces, respectively) at different occupancy levels. Bottom Right: χ_3_ RC after dFoCC or traditional refinement (blue and orange traces, respectively) at different occupancy levels. dFoCC result for accurately estimated occupancy is highlighted in red, while based on the educated guess of the quantum yield (QY) in black. Traditional refinement was performed with phenix.refine via Xtrapol8, and against extrapolated structure factors. (B) Final DED_c_ map for educated guess dFoCC refinement (N=3) superposed over dark-adapted structure (grey) and refined model (green). (C) Final DED_c_ for accurate occupancy dFoCC refinement (N=11), shown as in (B). Notice that both occupancy estimation strategies converge to the same CC value and with it, almost identical poses for E384. (D) Traditional refinement results for accurate occupancy refinement (N=11). Left: Computed DED_c_ based on phenix.refine results. Right: extrapolated density map based on amplitudes used by phenix.refine.
